# How are health-related behaviours influenced by a diagnosis of pre-diabetes? A meta-narrative review

**DOI:** 10.1186/s12916-018-1107-6

**Published:** 2018-07-27

**Authors:** Eleanor Barry, Trisha Greenhalgh, Nicholas Fahy

**Affiliations:** Nuffield Department of Primary Care Health Sciences, University of Oxford, Radcliffe Primary Care Building, Radcliffe Observatory Quarter Woodstock Road, Oxford, OX2 6GG UK

**Keywords:** Diabetes prevention, Socio-cultural influences, Risk perception, Systematic review

## Abstract

**Background:**

Several countries, including England, have recently introduced lifestyle-focused diabetes prevention programmes. These aim to reduce the risk of individuals with pre-diabetes developing type 2 diabetes. We sought to summarise research on how socio-cultural influences and risk perception affect people’s behaviour (such as engagement in lifestyle interventions) after being told that they have pre-diabetes.

**Methods:**

Using the RAMESES standards for meta-narrative systematic reviews, we identified studies from database searches and citation-tracking. Studies were grouped according to underlying theorisations of pre-diabetes. Following a descriptive analysis, the studies were synthesised with reference to Cockerham’s health lifestyle theory.

**Results:**

In total, 961 titles were scanned, 110 abstracts assessed and 35 full papers reviewed. Of 15 studies included in the final analysis, 11 were based on individual interviews, focus groups or ethnography and five on structured questionnaires or surveys. Three meta-narratives emerged. The first, which we called biomedical, characterised pre-diabetes as the first stage in a recognised pathophysiological illness trajectory and sought to intervene with lifestyle changes to prevent its progression. The second, which we called psychological, focused on the theory-informed study of the knowledge, attitudes and behaviours in people with pre-diabetes. These studies found that participants generally had an accurate perception of their risk of developing diabetes, but this knowledge did not directly lead to behavioural change. Some psychological studies incorporated wider social factors in their theoretical models and sought to address these through action at the individual level. The third meta-narrative we termed social realist. These studies conceptualised pre-diabetes as the product of social determinants of health and they applied sociological theories to explore the interplay between individual agency and societal influences, such as the socio-cultural context and material and economic circumstances. They recommended measures to address these structural influences on lifestyle choices.

**Conclusions:**

The study of pre-diabetes to date has involved at least three research disciplines (biomedicine, psychology and sociology), which up to now have operated largely independently of one another. Behavioural science and sociology are increasing our understanding of how personal, social, cultural and economic aspects influence health-related behaviours. An interdisciplinary approach with theoretically informed multi-level studies could potentially improve the success of diabetes prevention strategies.

**Trial registration:**

Prospero Registration Number: CRD42018088609.

**Electronic supplementary material:**

The online version of this article (10.1186/s12916-018-1107-6) contains supplementary material, which is available to authorized users.

## Background

In the UK, there are 4 million people diagnosed with diabetes, 95% of whom have type 2 diabetes mellitus (T2DM). This has a major impact on the health of the individuals through microvascular disease (diabetic retinopathy, diabetic nephropathy and neuropathy), macrovascular disease (heart attacks and strokes) and mental health problems [1]. T2DM has a huge financial impact on the National Health Service (NHS) with 10% of its budget being spent on treating diabetes. The total cost of diabetes (direct and indirect costs) is estimated to be £23.7 billion and is expected to rise to £39.8 billion by 2035/36 [1]. As a consequence, diabetes prevention has become a national health priority [2].

Current UK diabetes prevention policy is based on using probability scores to identify those at high risk of T2DM and offer them a blood test screen [3]. The term ‘pre-diabetes’ has been created to encapsulate all individuals who have abnormal glycaemic blood tests but have not reached diabetic thresholds. The aim of the at-risk categorisation is to identify, monitor and refer people to interventions or medical treatment to prevent the development of T2DM [3]. These interventions are based on randomised controlled trials, which show that lifestyle measures and medication can reduce diabetes incidence and delay diabetes onset in those with pre-diabetes [4, 5]. Another high-risk group is women with a history of gestational diabetes (GDM – diabetes developing during pregnancy); 70% of such women progress to T2DM within 10 years [6].

NHS England recently commissioned a national Diabetes Prevention Programme (DPP) [7] in which those identified as pre-diabetic are offered a lifestyle intervention programme. Patients diagnosed with pre-diabetes will generally experience no illness and may never go on to develop diabetes [8]. As part of the DPP, their weight, glycaemic control (HbA1c) and blood pressure are monitored annually [3]. Our recent quantitative systematic review [9] revealed that only about one-third of individuals identified by screening programmes as pre-diabetic actually attend intervention programmes. There is limited research on the reasons for this low uptake.

The emergence of diabetes prevention programmes directed at people at risk of diabetes raises important concerns of how this labelling alters a person’s health-related behaviour. Being invited to a lifestyle education programme may not automatically result in behavioural change. A diagnosis of pre-diabetes may increase motivation for individuals to change their behaviour, but it may also cause harm by inducing anxiety over a condition that may never develop [8, 9]. Lifestyles targeted by policy interventions are more than just behaviours; they are social practices that are socially and culturally shaped. Social practices develop to coordinate with daily routines (such as cooking, eating and family interactions) and cannot be meaningfully studied in isolation. They link with other social practices in bundles [10], creating a complex web of interdependent activity. Exploring the interactions between social practices (for example, through individual narrative interviews or ethnography) can help identify the wider determinants of health [10].

In this study, we sought to review the published literature on how a diagnosis of pre-diabetes influences behaviour, taking account of influences at both the individual and social levels. To cover as broad a range of the literature as possible, we sought a methodology that would allow us to combine both qualitative and quantitative studies from a range of different disciplines.

### Aim, research questions and objectives

The aim of the study was to inform diabetes prevention policies by exploring how individual perceptions and wider socio-cultural influences shape individual health behaviours in response to a pre-diabetes diagnosis (or equivalent).

The specific research questions were:

A. How do individuals with pre-diabetes understand what it means to be at risk of developing diabetes?
B. Do people believe that their wider socio-cultural environment will affect their ability to make lifestyle changes? If so, in what ways?
C. What are the implications of these findings for the design and delivery of diabetes prevention programmes?

Specifically, we sought to:

- Identify primary studies that had explored health-related behaviours in populations at risk of diabetes
- Develop a taxonomy of these studies in terms of their epistemological assumptions and methodological approaches
- Extract and analyse data on risk perception, health-related lifestyle changes and individual and socio-cultural influences on these
- Synthesise findings from studies across disciplines, using seemingly conflicting data to draw out higher-order insights
- Draw conclusions on implications for the design and refinement of diabetes prevention programmes

## Methods

The study was undertaken as part of a MSc in public health and as background to a doctoral study in which the experience of pre-diabetes will be studied qualitatively. As desk research, this review did not require research ethics approval. The work was part of a wider programme of research that included a quantitative systematic review of screening programmes for pre-diabetes and the efficacy of lifestyle interventions and metformin in reducing progression to T2DM [9].

### Choice of approach

An initial browsing search revealed that empirical studies on how pre-diabetes influences behaviour had been undertaken from multiple perspectives and published in a wide range of journals, resulting in at least two separate literatures with little dialogue between them. Both quantitative (survey) and qualitative (interview, focus group and ethnography) studies had been undertaken, with different underlying assumptions about what pre-diabetes was and how human behaviour should be studied. Seeking to embrace all previous research on the topic, we initially began a mixed-studies review as described by Pluye et al. [11].

However, it quickly became apparent that the key challenge in this review would be synthesising data from studies that had different ontological and epistemological starting points. For this reason, we adopted the meta-narrative approach originally proposed by Greenhalgh et al. and developed further as the RAMESES standards [12]. A meta-narrative review takes as its unit of analysis the research tradition, which is a linked set of studies from a common (but evolving) set of conceptual assumptions and theoretical approaches. Scientists in one research tradition tend to build on (and/or seek to refute) the work of previous scientists in the same tradition but pay less attention to researchers outside that tradition. The methodology aims to highlight—and generate higher-order data from—the contrasting ways in which different research traditions have studied the same topic.

Meta-narrative review is an interpretive review methodology based on six principles: pragmatism (when working with a large and heterogeneous literature, select sources that appear most relevant to a particular problem), pluralism (acknowledge and celebrate that different researchers have examined a topic in different ways), historicity (consider which earlier studies influenced which later ones), contestation (use conflicting findings to drive the search for richer explanations), reflexivity (critically examine your own assumptions, methods and emerging findings) and peer review (present emerging findings periodically to external audiences and take account of their feedback). It has much in common with other interpretive approaches such as hermeneutic review [13] and critical interpretive synthesis [14]. All these approaches share a desire to generate meaning; they favour sense-making and theory-building over methodological scoring systems and checklists.

### Search strategy

We sought to identify all studies relating to populations that explored individuals’ risk perceptions and/or the psychological, social, cultural and material influences on lifestyle change in pre-diabetes. There is international inconsistency in how to define pre-diabetes with different countries using different parameters and inclusion criteria in prevention programmes. To reflect this, we looked for studies that assessed the impact on health-related behaviours of informing people they were at risk. Such behaviours included attendance for testing, engagement with lifestyle programmes and changes in diet and activity levels. Peer-reviewed qualitative (semi-structured interview, focus group or ethnography) and quantitative (closed-item questionnaire and survey) studies were eligible. Research on populations with established diabetes and studies focusing exclusively on children were excluded.

Study identification was undertaken between May 2016 and August 2017. With guidance from a specialist librarian, searches of Medline, Pre-Medline and Embase were undertaken. The search strategy is shown in Additional file 1. The search terms (medical subject headings and free text) included ‘test’, ‘screening’, ‘pre-diabetes’, ‘impaired glucose tolerance’, ‘impaired fasting glucose’, ‘gestational diabetes’, ‘post-partum’, ‘ethnic groups qualitative research’, ‘social cultural’, ‘risk’ and ‘health related behaviours’. With a view to identifying papers in both biomedical and social science traditions, we undertook further searches of the Cochrane Database, International Bibliography of the Social Sciences and Web of Science. Citations of key papers were followed in Google Scholar to identify other relevant titles. Preliminary searches were performed in May 2016 with repeat searches for papers published in the last 12 months undertaken in June 2017. In addition, citations of all the key papers identified in our 2016 search were followed in Google Scholar in June 2017 to see if any new papers had cited them. A further paper was added to the data set following a recommendation from a peer reviewer. We manually extracted relevant titles from this data set and reviewed abstracts to identify papers for full review.

### Data extraction

Given the large number of studies identified in the initial searches, full text papers were assessed initially for relevance and were subject to rapid appraisal using the Critical Appraisal Skills Programme (CASP) checklist [15] (Additional file 2). Papers not meeting basic criteria for CASP (such as relevance to the review title) were excluded from further analysis.

For each study, data were extracted on four key questions for a meta-narrative review [12]: (a) How has the issue been conceptualised by researchers? (b) How has it been theorised (explicitly or implicitly)? (c) What methodological approaches have been used to study it? (d) What were the key findings? Papers that discussed ethical considerations as well as gaining ethical approval gained a point for their CASP score for this question.

### Quality assessment

Further assessment of the quality of the papers was undertaken using an adapted CERQual (Confidence in the Evidence from Reviews of Qualitative Research) framework [16], a tool designed to indicate the level of confidence in research findings. It was created in response to the increasing use of qualitative research in health-care institutions and health-care policy. The CERQual tool is not a technical checklist but gives an overall structure to a quality appraisal, leaving room for the reviewer’s interpretation of the evidence [16]. The four domains are methodological limitations, relevance, coherence and adequacy of data. The CERQual tool [17] was adapted using published literature on how to assess the methodology and quality of the studies [17]. Questions pertinent to the four domains as well as questions relevant to quantitative research were included in the checklist (for full details, see Additional file 3).

### Theoretical approach

It was evident from our data set that the studies identified sought to account for behavioural change through quite different types of explanation, some using psychological theories, some taking more sociologically informed perspectives and some not using any explicit theoretical framework at all.

To combine these different approaches, we selected Amartya Sen’s theoretical model, the health capabilities framework, which states that health outcomes in lifestyle-related diseases are the result of interaction between choices (human behaviour) and chances (the socio-cultural contexts that make some choices more feasible and meaningful than others in particular contexts) [18].

**Fig. 1:**
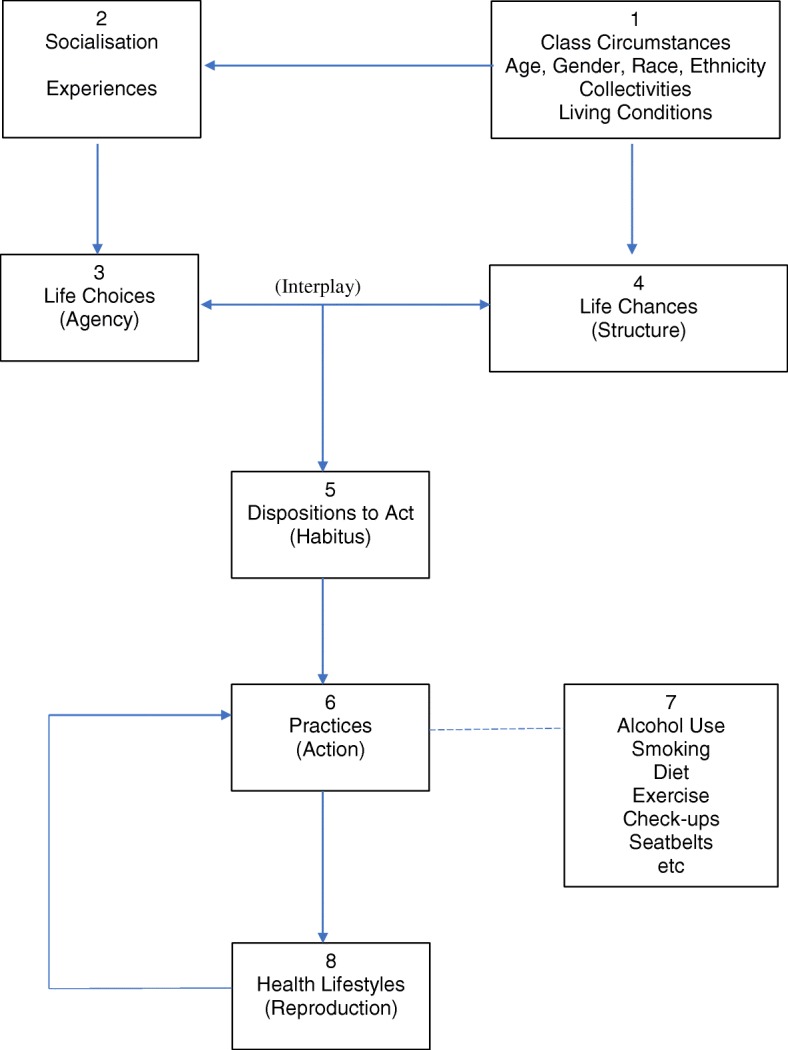
Health lifestyle theory [19]

Health lifestyle theory [19] builds on these principles, as illustrated in Fig. 1. Key to this theory is an exploration of the influences forming individual life choices and life chances. Central to this is Bourdieu’s notion of habitus, that is, the internal dispositions and tendencies that have been generated and shaped by particular socio-cultural experiences. In turn, the way people think and act (including the choices they make) also influences the wider socio-cultural environment and evolves over time [20].

The studies in our data set were mapped diagrammatically onto the healthy lifestyle framework to visualise the approach undertaken by the different studies, identifying which components of the theory the studies focused on.

### Synthesising the literature

We took an emergent approach, keeping the initial inclusion criteria broad and selecting papers by relevance to the review question. As the analysis developed, we selected further papers to test emerging theories. Microsoft Excel spreadsheets were used to aid data management. Using the iterative approach recommended for a meta-narrative review, we undertook a critical assessment of the literature, explored contradictory results, challenged authors’ interpretations and understanding of problems, and considered the strengths and limitations of the approaches taken. A line of argument incorporating the overarching similarities and differences in the perspectives between different research traditions was developed. These were mapped onto the healthy lifestyle framework.

## Results

### Search results

**Fig. 2:**
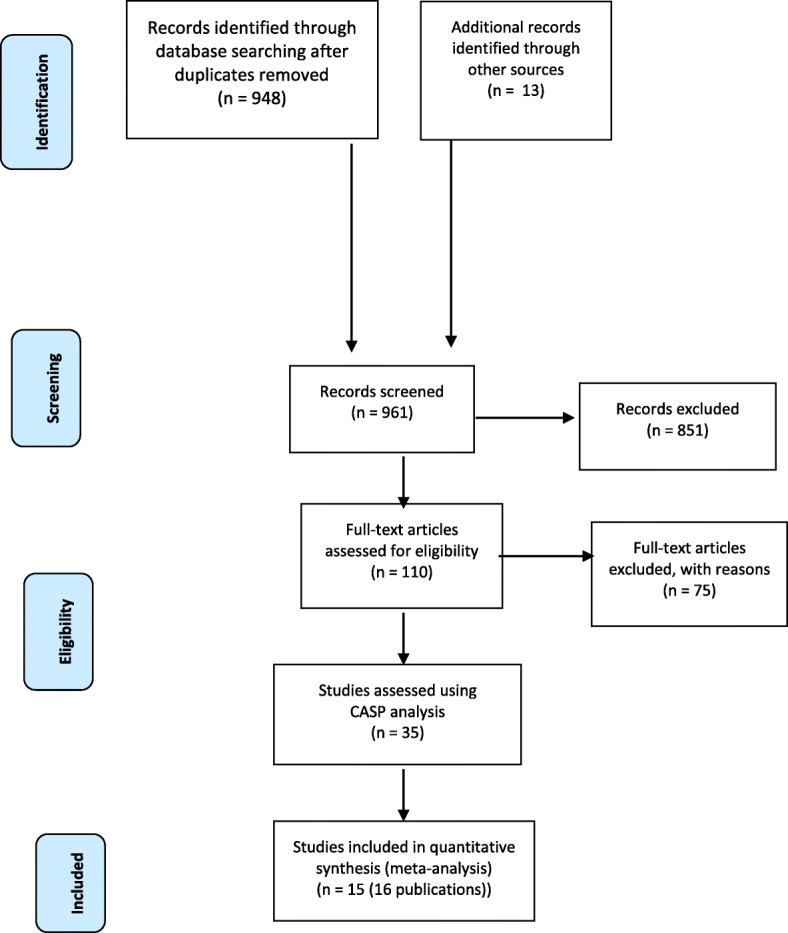
Flow chart

The study flowchart is shown in Fig. 2.

A large number of articles were identified through the search databases but the relevance of many of these to the review question was low. Citation-tracking identified a small number of highly relevant articles (50% of the final sample). In total, 35 publications underwent a full paper review and 15 studies were included in the final analysis. The final sample included four quantitative studies (using questionnaires and surveys), one mixed methods study and 10 qualitative studies (including 154 participants from individual interviews and 312 participants from focus groups). Seven studies recruited participants who were currently or previously enrolled in diabetes prevention trials or interventions.

The list of papers included in the study and their methods are shown in Table 1. Different papers addressed at least one of research questions set out by the review. The maximum CASP score was 10/10 and all papers scored at least 7/10. Reasons for exclusion included lack of relevance to the title of the review [21], participants not meeting our inclusion criteria (e.g. studies undertaken in people who did not have an elevated risk of diabetes or pre-diabetes) [22] and studies that focused exclusively on GDM and the not future risk of diabetes [23]. The CERQual tool was used to assess the quality of the papers included in the review. A score of 1.0 reflects that there were no major methodological flaws in a paper whereas a score of 0.1 signifies that there were several methodological flaws. Full details of the CASP and CERQual assessments can be found in Additional files 2 and 3.

**Table 1:** Summary of studies included

Author	Paper no	Research perspective	Study design	Study population	Theory or framework used	CASP score	CERQual score
Hindhede 2014 [29, 30]	1,2	Social realist	In-depth semi-structured interviews	10 individuals participating in intervention; focus groups with 14 clinicians	Bourdieu: habitus Giddens: agency/structure Weber: choices/chances	9	1
Greenhalgh 2015 [31]	3	Social realist	Group storytelling and in-depth narrative interviews	South Asian women with a history of GDM, 17 in focus groups and 28 individual narratives	Glass and McAtee’s axis of nested hierarchies influencing behaviours and disease risk Giddens: agency/structure Weber: choices/chances	9	1
Jallinoja 2008 [32]	4	Biomedical	Structured focus groups with pre-defined questions	30 individuals interviewed after a lifestyle intervention.	No explicit theoretical framework, though references to Giddens’s reflexivity and individuality and self-determination theory	9	0.5
Walker 2012 [28]	5	Psychological	Structured focus groups	29 people a year after a lifestyle intervention	Health action process approach (Schwarzer)	8	0.5
Troughton 2008 [36]	6	Biomedical	1:1 semi-structured interviews	15 participants, 40% with South Asian ethnicity	Leventhal’s self-regulatory model of illness behaviour referred to in discussion but not in analysis	8	0.1
Satterfield 2003 [35]	7	Biomedical	Open-ended focus groups	235 persons from a mixed US population	None	7	0.1
Tang 2015 [38]	8	Psychological	Semi-structured interviews	23 women with a history of GDM within the last year	Health belief model	9	0.5
Vlaar 2014 [27]	9	Psychological	Structured questionnaire (Likert scales)	535 people in a randomised controlled trial on diabetes prevention	Leventhal’s self-regulatory model of illness behaviour	9	1
Kim 2007 [24]	10	Psychological	Telephone or written survey	217 women of white ethnicity with a history of GDM	Health belief model	8	0.5
Jones 2011 [25]	11	Psychological	Quantitative survey with semi-structured interview	22 women with a history of GDM within the last 7 years.	Risk perception attitude framework	8	0.5
Morrison 2014 [33]	12	Biomedical	Semi-structured interviews	20 trial participants and four family volunteers	None	9	0.5
Penn 2015 [34]	13	Biomedical	Semi-structured interviews	15 intervention participants from a South Asian ethnic group	None (theoretical domains framework used in structure coding)	8	0.5
Kolb 2015 [26]	14	Psychological	60-item multi-choice survey	54 black or Hispanic women	Trans-theoretical model of stages of change	8	1
Morrison 2010 [39]	15	Biomedical	Cross-sectional analysis of national survey	1381 women with a history of GDM	None	9	1
Penn 2018 [37]	16	Biomedical	Semi-structured interviews and focus group as part of an evaluation	21 people with pre-diabetes undertaking DPP	None	9	1

*DPP* Diabetes Prevention Programme, *GDM* gestational diabetes

### Key research traditions

The studies reviewed revealed a range of assumptions about the nature of reality. Broadly speaking, they fell into three categories, each of which might be considered a meta-narrative [12]. All included studies could be aligned with one of these three meta-narratives, though there was some cross-fertilisation of ideas between traditions (e.g. when a paper in one tradition mentioned a different theoretical perspective in passing in the discussion).

The first category was what we called the biomedical meta-narrative. In this, T2DM was conceptualised in epidemiological terms as resulting from the interplay of antecedent risk factors and environmental causes. From this perspective, the perceptions of participants about how they understood diabetes and their possible actions were themselves analysed as risk factors and causes. These studies did not include theories (either psychological or sociological) as a major element of the analysis and proposed solutions in terms of individual behavioural change as a way of reducing one or more risk factors.

The second category was what we called the psychological meta-narrative. These studies drew on psychological theories to surface and analyse the perceptions of participants. Some of these studies took a cognitive approach (such as focusing on individual perceptions of risk and its relation to behaviour, or stages of change) [24–26]. Others took a social cognitive approach, incorporating participants’ perceptions of their social and cultural context within their analysis, such as through Leventhal’s self-regulatory model [27] of illness behaviour or the health action process approach [28]. In keeping with a psychological approach, though, these studies focused on the individual within a social context, rather than on the social context itself.

Our third category was what we called the social realist meta-narrative. These studies took a sociological approach, focusing on the social determinants of health, consisting of relevant aspects of the social, cultural and economic environments, which were seen as both shaping and constraining individual predisposition and behaviour. Some of these papers questioned the origins of the category pre-diabetes, viewing it as partly socially constructed and asking whose interests the diagnosis served [29]. These studies used sociological theories to explore the interplay between human behaviour and external social and material influences [29–31]. They framed solutions mainly in terms of addressing the wider social context in which disease (or pre-disease) develops.

Table 2 summaries the key findings from the studies by epistemological perspective. Additional file 4 gives a detailed description of authors’ perspectives and findings from individual studies.

**Table 2:** Three meta-narratives of pre-diabetes

Question	Biomedical	Psychological	Social realist
How has the problem been conceptualised by the authors?	Pre-diabetes is a biomedical condition that is a precursor for diabetes.	Pre-diabetes is an objective risk state. People require a perception of high risk and knowledge to change their lifestyles and reduce their diabetes risk. Social context has a role to play in changing behaviours within the individual.	Development of type 2 diabetes is a complex process influenced by multiple social, cultural and environmental factors. The term ‘pre-diabetes’ is (at least in part) a socially constructed and value-laden category that obscures these wider determinants.
	People can reduce their risk by changing their lifestyles in a prescriptive way.		
How has the problem been theorised?	Chronic disease develops in a linear fashion (genetic predisposition to risk state to established disease).	Psychological models of health-related behaviour (especially Leventhal’s self-regulatory model of illness behaviour and the health belief model).	Sociological models of the interaction between agency (individual behaviour and choices) and wider social influences (structure), especially Bourdieu’s notion of habitus (internal predispositions shaped by cultural experiences).
What methods have been used to research the problem?	Questionnaires and semi-structured focused interviews.	Semi-structured interview and focus group studies seeking data on psychological factors (attitudes, perceptions, concerns and barriers to change or engagement). Questionnaire studies of attitudes, stage of change, self-reported behaviours, risk assessment and disease knowledge.	Interviews and ethnographic studies seeking a rich picture of how wider social and cultural influences affect individual decision-making and action. Lifestyles are viewed as social practices with cultural meaning and moral worth.
What instruments have been used to measure key variables or influences?	Quantitative scales and questionnaires. Qualitative data from focus groups.	Quantitative scales and questionnaires. Qualitative data from focus groups.	Critical ethnography, analysis of individual narratives (e.g. of family life) and analysis of wider cultural storytelling narratives (e.g. of diaspora or oppression).
What are the main findings?	A diagnosis of pre-diabetes is sometimes (but not always) accepted and seen positively as prompting behavioural change.	People with pre-diabetes do not always perceive themselves at high risk of developing type 2 diabetes, even when they know the risk factors. Social context has an important role to play in changing lifestyles.	Perceptions and actions are socio-culturally framed.
			Lifestyle change is possible only when (and to the extent that) the individual’s social context, culture, and material and economic situation support particular behaviours.
What conclusions are drawn from the findings?	Diabetes prevention can be improved through individual lifestyle education. This should focus on improving knowledge.	Diabetes prevention can be improved through lifestyle change by increasing risk perception and knowledge. However, social context is an important determinant of individual behavioural change.	Diabetes prevention through individual lifestyle education will have limited impact unless wider socio-cultural, environmental and material influences are addressed.

In the next section, we describe the three key meta-narratives of pre-diabetes in the research literature before synthesising an overarching account of the condition using health lifestyles theory.

### Meta-narrative 1: pre-diabetes as a biomedical condition

#### The pre-diabetes diagnosis

The studies that took a biomedical perspective accepted pre-diabetes as a medical diagnosis and saw this as a precursor to T2DM. For example, Jallinoja et al. present pre-diabetes as an objective medical condition and summarise empirical evidence of the effectiveness of lifestyle interventions in its prevention [32].

Responses from the participants to the diagnosis of pre-diabetes differed between all the studies. Some described the condition as ‘being on the borderline of developing diabetes’ [33] and gave a strong incentive to engage in interventions and change lifestyles [34]. Some people welcomed the diagnosis of pre-diabetes and were pleased that it was ‘not yet diabetes’ [35]. In contrast, a UK community-based qualitative study found participants had ‘never heard of this pre-diabetes stuff’ [36]*,* rejecting the categorisation with ‘I cannot see that I have got, that I am pre-diabetic, because I am not a great sugary lover’ [36]*.* Some revealed confusion on how to prevent diabetes: ‘I want to prevent it if I can, and I do not know how. I am up in the air and hoping’ [36]*.* As a consequence of these findings, researchers introduced the term ‘non-diabetic hyperglycaemia’ as an alternative diagnostic label.

#### Socio-cultural influences

The biomedical studies focused almost exclusively on the individual to bring about behavioural change with varying degrees of focus on socio-cultural influences. For example, Jallinoja et al. explored how individuals change their lifestyles and to what extent they are autonomous in this [32]. The authors identified key themes of self-regulation, self-control, individualisation and autonomy. They summarised their findings by depicting three contrasting repertoires—hopeless, struggle and self-governing—depending on the individual’s perceptions and their ability to self-regulate. For example, participants classified as having a hopeless repertoire exhibited

‘some motivation to show that one went along with the rest of the group and as the sessions ended the individual became disengaged from lifestyle change pursuit, with fading out of the novel behaviours formed during the programme.’

In turn, these participants felt guilty, blaming themselves for this failure. In contrast, the participants classified as having a self-governing repertoire were able to self-monitor and self-govern and had self-control: ‘this is part of this life … and that you can control it… I now control this system in myself’ [32]. There is the implication that these behaviours can to some extent be influenced through education. However, the studies did not consider the social circumstances that might make a person with pre-diabetes feel more or less in control.

Many authors discussed ‘the importance of social influences, as well as social role and identity’ [34]. Satterfield et al. identified structural elements that have been shown to restrict life choices, such as environmental constraints (not enough parks and green spaces), economic constraints and lack of community support [35]. Others identified enablers to lifestyle change, such as person-centred advice from medical professionals and supportive family and friends [36]. Penn et al. also confirmed a number of influences on lifestyle change, such as embarrassment about size, cost of gym access and the emotional complexity of food intake [34]. Morrison et al. [33] identified cultural barriers and enablers to engaging in lifestyle change that determined the extent participants could adhere to such change. For example, dietary interventions did not resonate with international food preferences, creating barriers to lifestyle change:

‘Once a week they have children all come so we feel that the food should be much nicer according to the tradition and also children don’t like ordinary vegetables they fancy food like from McDonald’s so just to compete with that kind of food we try to make our old Indo-Pakistani dishes.’

Moreover, ‘food was described as a cultural representation of warmth’. Penn et al.’s [37] paper evaluating the NHS DPP identified difficulties faced by individuals when changes to behaviours (such as healthy eating) conflicted with social practices:

“When you go to somebody’s home and they’ve invited you in and they’ve prepared a meal for you, it’s very difficult to say, ‘I won’t eat that. I can’t eat that. I shouldn’t eat that.’” [37]

In summary, biomedical studies did appreciate the presence of wider social, cultural and economic influences on behavioural change. However, their focus at an individual level meant that these wider influences were documented as individual descriptions. These studies did not further analyse wider structural influences as objects of study using psychological or social theories. As a consequence, future research and policy recommendations focused on individual-level interventions.

### Meta-narrative 2: pre-diabetes from a psychological perspective

#### Pre-diabetes and risk

The psychological studies took an individual approach to studying pre-diabetes. These authors accepted the categorisation of pre-diabetes [28, 38] and did not question its establishment. Four psychological studies examined the effects of the diagnosis by asking participants about their perceived risk of developing T2DM. They calculated mathematical estimates of people’s risk perception, with regression analyses to see which risk factors (such as physical activity, weight, ethnicity, diet or family history) were associated with a perception of higher risk. They found that no individual risk factor was associated with a perception of high diabetes risk at a statistically significant level, although people with a family history of diabetes perceived themselves as having a higher risk of developing diabetes [25]. These studies reported that participants were able to identify risk factors associated with diabetes. For example, >90% of participants recognised that GDM was a risk factor for diabetes development [24, 25].

The ability to identify risk factors for diabetes did not always result in participants identifying themselves as being at high risk. Kim et al. [24] reported that over 90% of participants with a history of GDM were aware of the lifestyle changes required to prevent diabetes but only 16% thought they were at high risk of developing diabetes. In Vlaar et al.’s study of people with pre-diabetes, 72.5% identified South Asian ethnicity and 88.9% identified family history as risk factors for diabetes development [27]. Despite this, only 44% of respondents thought they were at high risk of developing diabetes. The participants in this study who attended a lifestyle intervention had a higher risk perception score and more knowledge of diabetes risk factors, compared to those who did not attend the intervention. However, this was not statistically significantly associated with attendance of the lifestyle intervention (odds ratio 1·76; 95% confidence interval 1·01–3·07) [27]. The studies that identified the disparity between understanding the high risk factors and a perception of low risk drew on Weinstein’s theory of unrealistic optimism, which suggests that people believe they are healthier than others because they focus on protective actions in their own case, but on risks when it comes to others [24, 27, 39].

Kolb et al.’s [26] study of American pre-diabetics also identified a high level of knowledge of diabetes risk factors and prevention strategies among the participants. This study reported that the participants felt it was their responsibility to change their behaviours and reported high motivation for changing their lifestyles. They identified the importance of social support in enabling lifestyle change but did not explore the role of wider contexts. Rather, this study used the trans theoretical model to analyse stages of behavioural change and was, thus, focused at the individual level.

#### Socio-cultural influences

Three of the psychological studies examined how socio-cultural contexts influence behavioural change within the individual and used social cognitive psychological theories to explain how these interacted with the individual.

Jones et al. used both questionnaires and semi-structured interviews of Indian American women with a previous history of GDM. They identified a discrepancy between the self-efficacy reported in questionnaires (which found that participants reported a high level of personal control in efforts to prevent diabetes) and the self-efficacy reported in semi-structured interviews. These women reported that they did not feel they could control their diabetes risk due to ‘American Indian cultures’ [25]. For example, one said:

‘Trying to actually practice it [behavioural change] in my home, yeah, it’s somewhat difficult, you know, because we’re all used to this lifestyle. And it’s a major change’. [25].

Food was identified as central to socialising within the American Indian community:

‘Everything revolves around food, and a lot of native peoples, that’s their highlight of any kind of social gathering is that you’ve got to have food to celebrate’.

Further to this:

‘Cooking like most of all Indians do; we fry everything, deep fry everything. Fry bread, fried potatoes, and we love it. That’s what was our meal; that’s what we were raised on.’ [25]

Many women in this study reported low confidence in preventing diabetes due to their family history of diabetes or ethnicity:

‘You know when you grow up and you just hear about those things, you know ‘Indians get diabetes.’ … It’s pounded in my head growing up.’ [25]

This study used a risk perception attitude framework in the analysis of the qualitative interviews, but this framework did not integrate an analysis of the wider social factors identified by participants.

Tang et al. [38] also explored how individual risk perception influenced engagement in behavioural change. Using qualitative interviews of women with a history of GDM, they found that the majority of participants perceived they had a high risk of developing diabetes. However, this did not act as a motivator for reducing their risk of diabetes and changing lifestyles. Children acted as a positive influence on behavioural change (women wanted to be healthy for them) but also were a barrier for change. Women reported difficulties accepting or accessing social support for childcare assistance, which meant they were unable to partake in lifestyle change such as exercise:

‘I don’t leave the children alone with non-family members and so that is difficult because if I am not exercising with them, with me, then I feel I have really leaned on my mother a lot for sitting so I don’t want to overdo it.’ [38]

Walker et al. [28], whilst focusing on the individual, explored how social support and community influences were critical in determining the success of lifestyle change strategies. Social support from partners and roles within the family unit were key in helping people change their lifestyles:

‘It’s difficult to change your own lifestyle if your partner and family don’t want to change theirs.’ [28]

In addition, life circumstances and community or group support were key in increasing physical activity. However, there were also community barriers such as hospitality and social acceptability of meals:

‘Households needed supplies of biscuits and cakes for visitors, while savoury scones or biscuits and cheese were healthy alternatives to cake for morning tea’. [28]

Social context and acceptability of lifestyle choices were seen as some of the greatest threats to sustained behavioural change. Walker et al. [28] analysed their results using the health action process approach model, which embeds the individual processes and stages of behavioural change within external contextual influences, such as family and community support. These were linked to the quantitative targets of the lifestyle intervention.

The psychological studies found that knowledge of diabetes risk factors and risk perception did not themselves lead to behavioural change. Qualitative psychological studies identified a number of structural barriers to lifestyle change and used psychological theories to explore how these were considered by individuals. Recommendations for further research and policy focused on individual-level interventions.

### Meta-narrative 3: pre-diabetes as a social realist construct

#### The pre-diabetes diagnosis

The two social realist papers framed their studies very differently. Whilst the individually focused studies implicitly assumed that individuals should be responsible for behavioural change, Hindhede et al.’s paper is a critique of how the medical diagnosis of pre-diabetes puts the full responsibility for behavioural change on the individual, thereby downplaying the importance of social and material circumstances in the development of T2DM [29]. Pre-diabetes is described as a ‘statistically constructed risk object’ [29], and linking risk to lifestyle leads to a portrayal of the individual being the greatest risk to their own health. Hindhede et al. report that this is a strategy for disciplinary power to monitor and govern individuals to achieve behavioural change [29]. Participants in their qualitative study interpreted the pre-diabetes diagnosis as an individual failing that needed a response through self-regulation. Fear of developing diabetes and the threat of medication by health-care professionals were all motivating influences in encouraging behavioural change. The Hindhede study also explored the views of health professionals who welcomed this categorisation. They saw pre-diabetes as a way to identify those in need of education, surveillance, self-management and behavioural change [29].

Greenhalgh et al. did not use the phrase ‘pre-diabetes’ because they deliberately adopted the words used by the study participants to describe their condition. Their study included participants who described themselves as having abnormal blood sugar levels following diabetes that came on in pregnancy [31]. Participants were anxious about the implications for subsequent pregnancies and the need for annual testing for diabetes. The participants’ fear of diabetes stemmed from the negative physical and emotional symptoms experienced in pregnancy alongside feelings of lack of control. This study found that health-related behavioural changes during pregnancy were performed primarily for the health of the unborn baby. These ante-natal efforts diminished after birth because the mother’s attentions were focused on the new baby and not themselves:

‘Right now, I’m just like whatever. It is just me. I am not worrying about another human being in my womb. It makes a big difference. Right now, I just need to get energy to take care of this guy right here.’ [31]

This study collected and analysed a number of narrative accounts identifying socio-cultural enablers and constraints influencing an individual’s risk perception and ability to act. This allowed the authors to undertake an in-depth exploration of the complexity behind diabetes development.

#### Socio-cultural influences

The social-orientated studies demonstrate how social and cultural factors have significant influences on lifestyle change. The Hindhede study included middle-class participants of white ethnicity and exhibited how social influences, such as identity, social capital, material circumstances, economic circumstances and self-regulation, acted as positive influences on lifestyle change [29]. The behavioural changes participants were being asked to adopt were not far removed from their existing lifestyles and could be readily incorporated into their existing routines. For example,

‘I liked it the minute I entered [the fitness club] … I cannot run on the treadmill, I only walk for like 10 min at a brisk trot.’ [29]

Another participant reported:

‘I have already exercised away much of the fat. I wake up every morning and cycle 11 km… I love it. It has become my way of life.’ [29]

In sum, these educated white European participants were able to incorporate a fitness identity within their existing lives and used this to enable their behavioural change.

This study also identified the importance of social capital as an enabler to lifestyle change, such as a supportive spouse: ‘I can ride a bike and that’s what we’ve been doing’ (pointing at his wife) [29]. However, social influences can also be negative:

‘There is peer pressure involved, you know…. When we are with friends, they do not accept a ‘no’, so you end up drinking that beer. It’s hard to lose weight’. [29]

A further influence identified in this study is the medicalisation of lifestyles through self-regulation (similar to the self-control group in the paper by Jallinoja et al. [32]). Participants use medical language and numerical values to monitor their progress and provide a source of motivation:

‘I didn’t like to look at myself in the mirror; it didn’t look good with that stomach. And twice a week, I measure my circumference and my weight. I write it down in my book here.’ [29]

Drawing on Bourdieu’s theory of habitus, Hindhede illustrates that lifestyle change is possible if the interventions being suggested are within material and economic reach of a participant and familiar to their existing social world.

In contrast, the study by Greenhalgh et al., which focused exclusively on South Asian women living in a deprived part of London, found that socio-cultural influences made health-related lifestyle change difficult [31]. Issues of identity, social capital, social context and material poverty were all important inhibitors of health-related lifestyle change. Deeply held cultural beliefs and practices (notably the heavy expectations of women’s domestic role) meant that women rejected exercise and fitness identities to keep their identity within traditional female roles. In this study, the same lifestyle changes that were accommodated readily by middle-class white Europeans (in the Hindhede paper) were depicted by these South Asian participants as unfamiliar, devoid of social meaning and lacking moral resonance.

In this study, friends and family depicted the kind of lifestyle changes recommended by doctors for people with pre-diabetes (dietary restriction and increased exercise levels) as unusual, inappropriate and even as a risk to their health. In the context of a group discussion on weight gain in pregnancy, for example, one woman said: ‘A lot of people advised me to eat this or eat that… so I followed their orders rather than just the doctors’ [31]*.* This, alongside the lack of social support, made it very difficult for these women to challenge peer advice and change their lifestyles. There were a number of constraints described by the cohort including scarcity of affordable healthy food, housing insecurity, cramped living conditions, high crime area and low walkability of the inner-city environment [31]. Participants reported that medical professionals presented unrealistic lifestyle strategies that were effectively impossible under the participants’ current socio-cultural and material constraints.

Whereas Hindhede’s middle-class white European participants sought to control their risk of developing diabetes through self-regulation, British South Asian participants in the Greenhalgh paper perceived their GDM to be out of control, and their accounts after the pregnancy reveal what a psychologist might describe as an external locus of control responsible for diabetes development (due to their perception that constraints on their lifestyle were insurmountable). As a consequence, these women tended to seek medication rather than lifestyle interventions to reduce their risk of T2DM.

Hindhede et al. and Greenhalgh et al. explored the dynamic interplay between agency and structure and how these interrelate to create the individual’s habitus and subsequent actions or inactions to change lifestyle. For example, the quote above about ‘following orders’ of family members and close friends illustrates that the social structure of peer pressure was often more influential than medical advice.

**Fig. 3:**
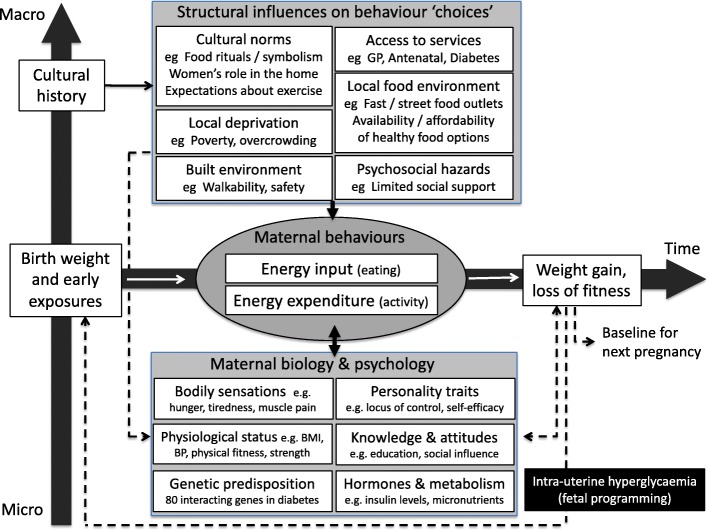
Schematic diagram adapted from Glass and McAttee showing the hierarchy of influences on diabetes risk, reproduced with permission from Greenhalgh et al. [31]

Greenhalgh et al. adapted Glass and McAtee’s axis of nested hierarchies, depicted by Fig. 3 [31]. It shows how medium- and longer-term socio-cultural influences alongside individual considerations influence behaviour and disease risk. The perpetual cycle of these influences accumulates over time to restrict the life chances of the individual and their family. This shows how wider social processes interrelate to shape lifestyles and the health-related behaviours of women and their families.

### Health lifestyle theory

**Fig. 4:**
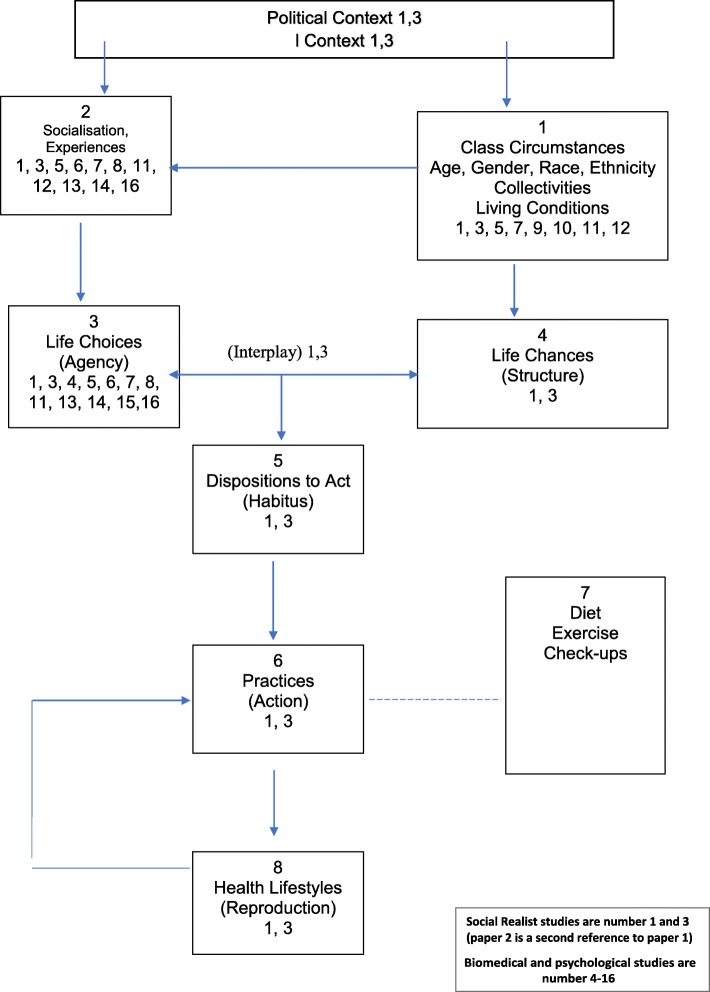
Mapping of studies to health lifestyle theory

Health lifestyle theory proved a good fit to the data and helped synthesise and map the approach taken by the studies included in this review [19]. Figure 4 is a depiction of this with each number corresponding to a study in Table 1. Added to the theory is an overriding box that recognises that there is a political context within which socialisation processes are created and structural disparities, such as class circumstances, are constructed. Again this was explored by Hindhede and Greenhalgh et al. [29–31] in their discussions and no other papers explored these influences. Figure 4 shows that the individually focused literature focuses on socialisation experiences and the life choices of individuals, reflecting the overriding emphasis for the individual to act. As we can see, all papers identify life choices as the area to target in diabetes prevention. Over half of the papers were able to identify socio-cultural structural factors that influenced lifestyle change. However, only two papers identify these as structural influences to be targeted in population-level diabetes-prevention strategies. Further to this, only two studies [29–31] using a social realist perspective looked at the interaction between agency and structure. They used the sociological theories of Bourdieu, Weber and Giddens [29–31] to interpret their findings and explain the practices and lifestyles depicted by their participants’ narratives.

## Discussion

### Principal findings

This meta-narrative systematic review explored how socio-cultural influences and risk perception contribute to health-related behaviours. The studies fell on an epistemological continuum from strong interpretivist to strong positivist approaches. For clarity, three main research traditions were identified from the literature.

The studies taking a biomedical perspective accepted the pre-diabetes categorisation. An implicit assumption in this labelling is that the participants will act linearly to reduce their risk of diabetes. This places the emphasis on the individual to act and assumes that people are free from everyday demands and constraints and can rationally adapt their lives. Social realists critique the biomedical use of risk as being void of context and reductionist [40]. Some studies outlined the importance of personal and social structural influences on behavioural change. However, none of these explored the complexity of the condition or how structural influences are incorporated into the individual as part of the decision-making process. This draws attention away from population-level strategies and as a consequence, the authors’ discussions focus on individual life choices with the emphasis on the individual’s agency to reduce their own risk of diabetes.

Psychological studies also took an individual-level approach to exploring knowledge on diabetes risk factors, risk perception and whether these led to behavioural change. Risk perception was not associated with behavioural change and this was explored using cognitive psychological theories. Qualitative studies identified a number of social barriers to lifestyle change, such as peer, family and community support. The social cognitive psychological theories used in these papers analysed how these contextual influences affect the individual regarding behavioural change and intervention behaviour targets. However, none of the psychology studies discussed the contextual barriers as objects of study or as intervention targets in further research [34, 35].

Social realist studies, in contrast, challenged the pre-diabetes risk diagnosis as a construct that could be applied unproblematically to individuals. They used social theory to explore how agency and structure interrelate to create dispositions to act and lifestyle decisions. One study, of middle-class white Europeans with high social capital, contrasted with another, of deprived South Asians with low social capital. The main themes influencing lifestyle change in these studies included cultural obligations, identity, self-control, self-surveillance, social capital, and economic and material circumstances. Lifestyle interventions were more likely to be successful if aligned to existing reality and if an individual has the personal and material resources to support change. However, for many, overwhelming structural influences will restrict their life chances, preventing any dispositions to act.

However, the social realist papers did not measure or theorise about individual psychological constructs such as self-efficacy and empowerment or draw on psychological theories in their analysis of the thoughts and actions of individuals. Had they done so, they may have identified psychological factors that help to overcome the structural influences for their choices. Hindhede [29] does not mention any psychological influences within the sociological paradigm. Greenhalgh’s [31] study references an ‘external locus of control’ in which individuals feel that diabetes development is out of their control, but did not draw on relevant psychological theory to situate that construct. It is presumed that this is due to overwhelming socio-cultural influences. A social realist analysis could be enhanced by the use of formal psychological frameworks to consider issues as self-efficacy and locus of control.

Mapping the studies onto the health lifestyle framework shows that the vast majority of studies focus on individual life choices as a way to change health-related behaviours. Neglecting the rest of the pathways means that current diabetes prevention interventions are unlikely to reduce the overall burden of disease. The mapping of the studies onto the health lifestyle framework shows that the majority of studies funded take an individual-level perspective. As a consequence, this limits the evidence available for policy use, perpetuating individual-level solutions to diabetes prevention.

### Comparison to other systematic reviews

Three qualitative literature reviews explore the experiences of those with pre-diabetes. The first was a review undertaken by O’Reilly et al. that focused on T2DM prevention in those with a history of GDM. This review identified international inconsistencies in diabetes prevention guidelines and explored qualitative work that identified many structural barriers to engagement with screening, interventions and breastfeeding. This team concluded that the solutions lie in individual-level interventions based on educating participants [41].

Shaw et al. undertook a systematic review of studies exploring patient experience of cardiovascular and diabetes prevention initiatives, such as NHS Health Checks [42]. The theoretical domains framework was used in the analysis of the qualitative literature, focusing on psychological behaviour models, which place the emphasis on the individual to act. However, the authors also add themes of context and social influence into their analysis, recognising the importance of upstream entry points for action. They conclude that national population-level policy is needed to support individual interventions.

Youngs et al. explore the impact of the pre-diabetes diagnosis on behavioural change [43]. They undertook a descriptive review of qualitative studies, quantitative studies and analysis reports. Many of the studies included in our review were also included in their review. However, their review strongly supports a biomedical approach to diabetes prevention, concluding that more work needs to be done to ‘innovatively’ increase people’s knowledge, self-belief and self-efficacy. One interpretation of their findings and conclusions is that if you look only for individual-level influences, you will find only solutions at that level.

This review increases both the breadth and depth of the understanding of how people change or do not change their behaviours following a diagnosis of pre-diabetes. Socio-cultural influences and risk perception play important roles in health-related behavioural change. It has illustrated how the epistemological perspective of authors has a large impact on how research questions are framed, what methodologies and data collection tools are used and how the data are interpreted. Two epistemological positions were identified in this review: the individual perspective and the social realist perspective. Findings from this review show that individual framings produce individual solutions and interpretivist sociological framings produce upstream solutions that are harder to implement. The social realist papers give further understanding as to why people respond and behave the way they do. Tensions between agency and structure show that even if people do want to change, increasing a participant’s knowledge and risk understanding is unlikely to result in health-related behavioural changes if they are subject to overwhelming structural factors. Bourdieu’s theory of habitus show that people are able to adapt their behaviours only if the interventions relate to their social environment and are within their material reach.

### Meaning and implications for public health and health policy

This systematic review has shown the wealth of knowledge and insight that can be obtained by public health social research. Current diabetes prevention policies focus on trial-based research, which lacks reflexivity and appreciation of the complex social mechanisms underlying the development of diabetes. Qualitative work that explores the construction of socio-cultural influences, how they contribute to the complexity of diabetes pathogenesis and structural barriers to lifestyle change are overlooked by diabetes prevention policies. By using academic work from different epistemological perspectives, we can gain a greater understanding of the complexity within which public health initiatives are exercised and insights into why these are or are not translated in practice. Here we have shown that authors taking an individual-level approach identify structural elements influencing behavioural change but have not made recommendations based on these.

Despite extensive knowledge of the wider influences constraining individual health behaviour, public health research has largely focused on individual statistical, psychological and economic models, which naturally lead to individualist solutions to diabetes prevention [10]. These paradigms are currently informing evidence-based policy with the assumption that what people do is divorced from society [10]. This creates a linear and oversimplified approach to T2DM prevention. Blue et al. [10] have called for a paradigm shift in how we ‘define, frame and evaluate behavioural change’ using a societal perspective. Swinburn [44] and Cypress [45] have called for greater attention to wider upstream structural factors to increase the life chances of the population. Taking this further, Green [46] and Rutter [47] et al. have called for a complex systems approach to multi-causal problems (such as diabetes), which require more than focusing on single interventions but rather ‘multiple elements in many systems’ [44]. These may have small effects on individuals, but may create large changes at a population level [44].

### Strengths and limitations of the review

This is a comprehensive meta-narrative systematic review of the literature that applied interpretive analytic methods to explore the role of socio-cultural influences and risk perception on health-related behaviours in diabetes prevention. This is the only systematic review to have explored the epistemological perspectives of studies, which it used to analyse the data with a new innovative approach for a mixed study review. We used CASP and an adapted CERQual tool for data extraction and quality assessment. We found these tools to be very helpful and did not think they restricted the interpretative approach of this review.

The use of two paradigms is an oversimplification and in reality, these studies fall on a continuum from a strong interpretivist perspective to a strong positivist perspective. There were also limitations to the primary studies. Seven of the studies recruited participants from trial settings, who are more likely to be motivated to create change and have a higher level of health literacy and fewer co-morbidities, and therefore, they are unlikely to be representative of the wider population [48]. In addition, some of the papers are almost 10 years old and discuss theories that may now be out of date. Additionally, there were no papers undertaken in a population at the time of diagnosis. Studies retrospectively explored how the diagnosis influenced health-related behaviours (e.g. Hindhede, Morrison and Troughton). Thus, there is a gap in the literature.

### Recommendations for future research

With the introduction of the NHS DPP in the UK, the first avenue for future work is expanding the qualitative work discussed in this paper. Only two studies took an interpretivist approach, which represents a gap in the literature. An ethnography taking an interpretivist perspective to explore the lived experience of pre-diabetics and whether this influences health-related behaviours may expand the knowledge base of existing studies. Undertaking an ethnography may also allow the mapping of the complex health system within which diabetes develops. Understanding this complexity will allow for interventions targeted at multiple elements within the system, rather than just the individual, which may provide greater scope for interpretivist perspectives within biomedical academic work. Increasing the dialogue between epidemiological, psychological and sociological perspectives to investigate systematic structural approaches and reviewing upstream processes may enrich policy recommendations [49]. A multi-disciplinary primary prevention strategy may enhance the current individualist research paradigm by exploring population-level approaches, and framing behaviours as social practices rather than individual choice [10]. These strategies may help to reduce the burden of many related long-term conditions, such as obesity, diabetes and cardiovascular disease.

## Conclusion

Socio-cultural influences and risk perception play important roles in determining whether the individual can adapt their health-related behaviours in response to being told that they have pre-diabetes. For those with structural influences that support behavioural change, such as material resources and positive social support, increasing their risk perception may be successful in leading to health behavioural change. However, those who have overwhelming socio-cultural structural inhibitors, such as poor housing, low material wealth, unsupportive environments and conflicting cultural influences, are unlikely to change their behaviours despite increasing their risk perception. This may explain the high attrition rates in engagement in interventions in deprived, ethnically diverse populations. Therefore, placing the onus entirely on individual agency to act is unlikely to have an impact on diabetes incidence. The development of a multi-faceted approach with emphasis on wider upstream structural influences to increase life chances may be needed to reduce the burden of disease. Wider involvement of interdisciplinary psychological and sociological perspectives in health policy construction may help to provide a greater understanding of the complexity of the conditions they are trying to prevent and improve the understanding of these in real-world settings.

## Additional files

Additional file 1: Search strategy. (DOCX 15 kb)

Additional file 2: Full CASP analysis. (DOCX 16 kb)

Additional file 3: CERQual analysis. (DOCX 21 kb)

Additional file 4: Detailed summaries of study findings. (DOCX 18 kb)

## Abbreviations

CASP: Critical Appraisal Skills Programme
CERQual Framework: Confidence in the Evidence from Reviews of Qualitative research Framework
DPP: Diabetes Prevention Programme
GDM: Gestational diabetes
NHS: National Health Service
NIHR: National Institute for Health Research
T2DM: Type 2 diabetes mellitus

## References

[1] Diabetes UK. Facts and stats. 2016. https://www.diabetes.org.uk/resources-s3/2017-11/diabetes-key-stats-guidelines-april2014.pdf

[2] NHS England. NHS five year forward view; 2014. https://www.england.nhs.uk/wp-content/uploads/2014/10/5yfv-web.pdf

[3] National Institute for Health and Care Excellence (NICE). Type 2 diabetes: prevention in people at high risk. NICE Guideline PH 38: NICE; 2012.

[4] Public Health England (PHE). A systematic review and meta-analysis assessing the effectiveness of pragmatic lifestyle interventions for the prevention of type 2 diabetes mellitus in routine practice. London; 2015. https://www.gov.uk/government/publications/diabetes-prevention-programmes-evidence-review

[5] Diabetes Prevention Program Research Group (2009). 10-year follow-up of diabetes incidence and weight loss in the diabetes prevention program outcomes study. Lancet.

[6] Bellamy L, Casas J-P, Hingorani AD, Williams D (2009). Type 2 diabetes mellitus after gestational diabetes: a systematic review and meta-analysis. Lancet.

[7] National Institute for Health Research (NIHR). Let’s prevent diabetes through education NIHR2016. Available from. https://www.nihr.ac.uk/news/lets-prevent-diabetes-through-education/4886.

[8] Barry E, Roberts S, Finer S, Vijayaraghavan S, Greenhalgh T (2015). Time to question the NHS diabetes prevention programme. BMJ.

[9] Barry E, Roberts S, Oke J, Vijayaraghavan S, Normansell R, Greenhalgh T (2017). Efficacy and effectiveness of screen and treat policies in prevention of type 2 diabetes: systematic review and meta-analysis of screening tests and interventions. BMJ.

[10] Blue S, Shove E, Carmona C, Kelly MP (2014). Theories of practice and public health: understanding (un)healthy practices. Crit Public Health.

[11] Pluye P, Gagnon MP, Griffiths F, Johnson-Lafleur J (2009). A scoring system for appraising mixed methods research, and concomitantly appraising qualitative, quantitative and mixed methods primary studies in mixed studies reviews. Int J Nurs Stud.

[12] Wong G, Greenhalgh T, Westhorp G, Buckingham J, Pawson R (2013). RAMESES publication standards: realist syntheses. BMC Med.

[13] Barnett-Page E, Thomas J (2009). Methods for the synthesis of qualitative research: a critical review. BMC Med Res Methodol.

[14] Dixon-Woods M, Cavers D, Agarwal S, Annandale E, Arthur A, Harvey J (2006). Conducting a critical interpretive synthesis of the literature on access to healthcare by vulnerable groups. BMC Med Res Methodol.

[15] Critical Appraisal Skills Programme (CASP) (2017). CASP CHECKLISTS: CASP UK.

[16] Lewin S, Glenton C, Munthe-Kaas H, Carlsen B, Colvin CJ, Gulmezoglu M (2015). Using qualitative evidence in decision making for health and social interventions: an approach to assess confidence in findings from qualitative evidence syntheses (GRADE-CERQual). PLoS Med.

[17] Greenhalgh T (1997). How to read a paper: assessing the methodological quality of published papers. BMJ.

[18] Weaver RR, Lemonde M, Payman N, Goodman WM (2014). Health capabilities and diabetes self-management: the impact of economic, social, and cultural resources. Soc Sci Med.

[19] Cockerham WC (2005). Health lifestyle theory and the convergence of agency and structure. J Health Soc Behav.

[20] Lupton D, Lupton D (2013). Sociology and Risk. Risk.

[21] Grigsby-Toussaint DS, Jones A, Kubo J, Bradford N (2015). Residential segregation and diabetes risk among latinos. Ethn Dis.

[22] Gele AA, Torheim LE, Pettersen KS, Kumar B (2015). Beyond culture and language: access to diabetes preventive health services among Somali women in Norway. J Diabetes Res.

[23] Kaptein S, Evans M, McTavish S, Banerjee AT, Feig DS, Lowe J (2015). The subjective impact of a diagnosis of gestational diabetes among ethnically diverse pregnant women: a qualitative study. Can J Diabetes.

[24] Kim C, McEwen LN, Piette JD, Goewey J, Ferrara A, Walker EA (2007). Risk perception for diabetes among women with histories of gestational diabetes mellitus. Diabetes Care.

[25] Jones EJ, Appel SJ, Eaves YD, Moneyham L, Oster RA, Ovalle F (2012). Cardiometabolic risk, knowledge, risk perception, and self-efficacy among American Indian women with previous gestational diabetes. J Obstet Gynecol Neonatal Nurs.

[26] Kolb JM, Kitos NR, Ramachandran A, Lin JJ, Mann DM (2014). What do primary care prediabetes patients need? A baseline assessment of patients engaging in a technology-enhanced lifestyle intervention. J Bioinform Diabetes.

[27] Vlaar EM, Nierkens V, Nicolaou M, Middelkoop BJ, Stronks K, van Valkengoed IG (2015). Risk perception is not associated with attendance at a preventive intervention for type 2 diabetes mellitus among south Asians at risk of diabetes. Public Health Nutr.

[28] Walker C, Hernan A, Reddy P, Dunbar JA (2012). Sustaining modified behaviours learnt in a diabetes prevention program in regional Australia: the role of social context. BMC Health Serv Res.

[29] Hindhede AL (2014). Prediabetic categorisation: the making of a new person. Health, Risk & Society.

[30] Hindhede AL, Aagaard-Hansen J (2014). Risk, the prediabetes diagnosis and preventive strategies: critical insights from a qualitative study. Crit Public Health.

[31] Greenhalgh T, Clinch M, Afsar N, Choudhury Y, Sudra R, Campbell-Richards D (2015). Socio-cultural influences on the behaviour of south Asian women with diabetes in pregnancy: qualitative study using a multi-level theoretical approach. BMC Med.

[32] Jallinoja P, Pajari P, Absetz P (2008). Repertoires of lifestyle change and self-responsibility among participants in an intervention to prevent type 2 diabetes. Scand J Caring Sci.

[33] Morrison Z, Douglas A, Bhopal R, Sheikh A, Trial I (2014). Understanding experiences of participating in a weight loss lifestyle intervention trial: a qualitative evaluation of south Asians at high risk of diabetes. BMJ Open.

[34] Penn L, Dombrowski SU, Sniehotta FF, White M (2014). Perspectives of UK Pakistani women on their behaviour change to prevent type 2 diabetes: qualitative study using the theory domain framework. BMJ Open.

[35] Satterfield DL, May T, Bowman JE, Alfaro-Correa BA, Benjamin A, Stankus C, Learning M. From listening: common concerns and perceptions about diabetes prevention among diverse American populations. J Public Health Manag Pract. 2003:S56–63. 14677332

[36] Troughton J, Jarvis J, Skinner C, Robertson N, Khunti K, Davies M (2008). Waiting for diabetes: perceptions of people with pre-diabetes: a qualitative study. Patient Educ Couns.

[37] Penn L, Rodrigues A, Haste A, Marques MM, Budig K, Sainsbury K (2018). NHS diabetes prevention Programme in England: formative evaluation of the programme in early phase implementation. BMJ Open.

[38] Tang JW, Foster KE, Pumarino J, Ackermann RT, Peaceman AM, Cameron KA (2015). Perspectives on prevention of type 2 diabetes after gestational diabetes: a qualitative study of Hispanic, African-American and white women. Matern Child Health J.

[39] Morrison MK, Lowe JM, Collins CE (2010). Perceived risk of type 2 diabetes in Australian women with a recent history of gestational diabetes mellitus. Diabet Med.

[40] Nettleton S (2004). The emergence of e-scaped medicine?. Sociology.

[41] O'Reilly SL (2014). Prevention of diabetes after gestational diabetes: better translation of nutrition and lifestyle messages needed. Healthcare (Basel).

[42] Shaw RL, Holland C, Pattison HM, Cooke R (2016). Patients’ perceptions and experiences of cardiovascular disease and diabetes prevention programmes: a systematic review and framework synthesis using the theoretical domains framework. Soc Sci Med.

[43] Youngs W, Gillibrand WP, Phillips S (2016). The impact of pre-diabetes diagnosis on behaviour change: an integrative literature review. Pract Diabetes.

[44] Swinburn BA, Sacks G, Hall KD, McPherson K, Finegood DT, Moodie ML (2011). The global obesity pandemic: shaped by global drivers and local environments. Lancet.

[45] Cypress M (2004). Looking upstream. Diabetes Spectr.

[46] Salway S, Green J (2017). Towards a critical complex systems approach to public health. Crit Public Health.

[47] Rutter H, Savona N, Glonti K, Bibby J, Cummins S, Finegood DT, et al. The need for a complex systems model of evidence for public health. Lancet. 2017;390(10112):2602–04. 10.1016/S0140-6736(17)31267-9 28622953

[48] Kennedy-Martin T, Curtis S, Faries D, Robinson S, Johnston J (2015). A literature review on the representativeness of randomized controlled trial samples and implications for the external validity of trial results. Trials.

[49] Nichter M, Nichter M (2008). Representations that frame health and development policy. Global Health: why cultural perceptions, social representations and biopolitics matter.

