# Supplementary material for: How are health-related behaviours influenced by a diagnosis of pre-diabetes? A meta-narrative review
Source: BMC Med. 2018 Jul 27;16:121. doi: 10.1186/s12916-018-1107-6 (PMC6062879; doi:10.1186/s12916-018-1107-6)
Supplement: Supplementary file 1 — Search strategy. (DOCX 15 kb) [file 12916_2018_1107_MOESM1_ESM.docx]

## Additional file 1: Search Strategy

Database: Ovid MEDLINE(R) <1946 to May Week 1 2016>, Embase <1974 to 2016 Week 19>

Search Strategy:

--------------------------------------------------------------------------------

1 health behavior/ or patient compliance/ or patient dropouts/ or treatment refusal/ (274453)

2 health behavior/ or attitude to health/ or behavioral risk factor surveillance system/ or health belief/ or health belief model/ or risk reduction/ (331907)

3 (complian$ adj3 patient$).mp. [mp=ti, ab, ot, nm, hw, kf, px, rx, ui, tn, dm, mf, dv, kw] (182432)

4 (complian$ adj3 participant$).mp. [mp=ti, ab, ot, nm, hw, kf, px, rx, ui, tn, dm, mf, dv, kw] (879)

5 health behavio?r$1.mp. [mp=ti, ab, ot, nm, hw, kf, px, rx, ui, tn, dm, mf, dv, kw] (102804)

6 (health adj3 attitude$).mp. [mp=ti, ab, ot, nm, hw, kf, px, rx, ui, tn, dm, mf, dv, kw] (388879)

7 ((patient$ or participant$) adj3 (dropout$ or dropping out)).mp. [mp=ti, ab, ot, nm, hw, kf, px, rx, ui, tn, dm, mf, dv, kw] (9080)

8 ((health or healthy or healthily) adj3 (eating or diet)).mp. [mp=ti, ab, ot, nm, hw, kf, px, rx, ui, tn, dm, mf, dv, kw] (22004)

9 (lifestyle or life style).mp. [mp=ti, ab, ot, nm, hw, kf, px, rx, ui, tn, dm, mf, dv, kw] (237432)

10 health promoting behavio?r$.mp. [mp=ti, ab, ot, nm, hw, kf, px, rx, ui, tn, dm, mf, dv, kw] (1593)

11 health related behavio?r$.mp. [mp=ti, ab, ot, nm, hw, kf, px, rx, ui, tn, dm, mf, dv, kw] (5108)

12 ((patient$ or participant$) adj3 refus$).mp. [mp=ti, ab, ot, nm, hw, kf, px, rx, ui, tn, dm, mf, dv, kw] (17404)

13 risk reduction behavior/ (76102)

14 or/1-13 (980462)

15 Prediabetic State/ (15856)

16 impaired glucose tolerance/ (22103)

17 Prediabetic state$.mp. (5602)

18 Pre-diabetic state$.mp. (331)

19 prediabet$.mp. (13420)

20 pre-diabet$.mp. (4637)

21 impaired glucose tolerance.mp. (35662)

22 impaired fasting glucose.mp. (7453)

23 non-diabetic hyperglyc?emia.mp. (67)

24 Diabetes, Gestational/ (16880)

25 gestational diabetes.mp. (22740)

26 (risk$ adj4 diabet$).mp. [mp=ti, ab, ot, nm, hw, kf, px, rx, ui, an, tn, dm, mf, dv, kw] (69049)

27 hyperglyc?emi$.mp. (147731)

28 or/15-27 (269809)

29 qualitative research/ (62767)

30 qualitative.mp. (341449)

31 exp "Surveys and Questionnaires"/ (776288)

32 exp questionnaire/ (1262689)

33 (survey$1 or questionnaire$ or focus group$1 or interview$).mp. [mp=ti, ab, ot, nm, hw, kf, px, rx, ui, an, tn, dm, mf, dv, kw] (2880036)

34 or/29-33 (3401022)

35 social behavior/ or sociological factors/ or social class/ or social conditions/ or social conformity/ or social control, informal/ or social distance/ or social environment/ or "social determinants of health"/ or hierarchy, social/ or social identification/ or social isolation/ or social marginalization/ or social medicine/ or social norms/ or social responsibility/ or social participation/ or social perception/ or public policy/ (600145)

36 culture/ or acculturation/ or cultural characteristics/ or cultural diversity/ or cultural evolution/ or ethnology/ or medicine, traditional/ or anthropology, medical/ (234643)

37 anthropology, cultural/ or exp cross-cultural comparison/ or cultural competency/ or cultural deprivation/ or ethnopsychology/ (132357)

38 (cultural or culture).mp. (2058293)

39 cultures.mp. (574483)

40 acculturation.mp. (10892)

41 (ethnolog$ or ethnic).mp. (298811)

42 anthropolog$.mp. (93691)

43 (social or society or sociolog$ or societal).mp. (3467369

44 exp Religion/ (114237)

45 (religio$ or belief$).mp. [mp=ti, ab, ot, nm, hw, kf, px, rx, ui, an, tn, dm, mf, dv, kw] (239256)

46 or/35-45 (6061184)

47 14 and 28 and 34 and 46 (1494)

48 remove duplicates from 47 (1157)

49 limit 48 to english language (1118)

50 conference.pt. (2968101)

51 49 not 50 (825)
