# Supplementary material for: How are health-related behaviours influenced by a diagnosis of pre-diabetes? A meta-narrative review
Source: BMC Med. 2018 Jul 27;16:121. doi: 10.1186/s12916-018-1107-6 (PMC6062879; doi:10.1186/s12916-018-1107-6)
Supplement: Supplementary file 2 — Full CASP analysis. (DOCX 16 kb) [file 12916_2018_1107_MOESM2_ESM.docx]

## Additional file 2: Full CASP Analysis

| **Author** | Paper no | Was there a clear statement of aims of the research | Was the methodology appropriate? | Was the research design appropriate to address the aims of the research? | Was the recruitment strategy appropriate to the aims of the research? | Was the data collected in such a way that addressed the research issue? | Has the relationship between the researcher and the participant been adequately considered? | Have ethical considerations been taken into account? | Was the data analysis sufficiently rigorous? | Is there a clear statement of findings? | Is the research valuable? |
| --- | --- | --- | --- | --- | --- | --- | --- | --- | --- | --- | --- |
| Hindhede 2014 | 1 and 2 | y | y | y | y | y | N | y | y | Y | y |
| Greenhalgh 2015 | 3 | Y | Y | Y | Y | Y | N | Y | Y | Y | y |
| Jallinoja 2008 | 4 | Y | Y | Y | Y | Y | N | Y | Y | Y | y |
| Walker 2012 | 5 | Y | Y | Y | Y | Y | N | Y | N | Y | y |
| Troughten 2008 | 6 | Y | Y | Y | Y | Y | N | Y | N | Y | Y |
| Satterfields 2003 | 7 | Y | Y | Y | N | Y | N | Y | N | Y | Y |
| Tang 2015 | 8 | Y | Y | Y | Y | Y | N | Y | Y | Y | Y |
| Vlaar 2014 | 9 | y | y | y | y | y | N | Y | Y | Y | Y |
| Kim 2007 | 10 | Y | Y | Y | Y | Y | N | N | Y | Y | Y |
| Jones 2011 | 11 | Y | Y | Y | Y | Y | N | N | Y | Y | Y |
| Morrison Z 2014 | 12 | Y | Y | Y | Y | Y | N | Y | Y | Y | Y |
| Penn 2015 | 13 | Y | Y | Y | Y | Y | N | N | Y | Y | Y |
| Kolb 2015 | 14 | Y | Y | Y | Y | Y | N | N | Y | Y | Y |
| Morrison 2009 | 15 | Y | Y | Y | Y | Y | N | Y | Y | Y | Y |
| Penn 2018 | 16 | Y | Y | Y | Y | Y | N | Y | Y | Y | Y |
