# Supplementary material for: How are health-related behaviours influenced by a diagnosis of pre-diabetes? A meta-narrative review
Source: BMC Med. 2018 Jul 27;16:121. doi: 10.1186/s12916-018-1107-6 (PMC6062879; doi:10.1186/s12916-018-1107-6)
Supplement: Supplementary file 3 — CERQual analysis. (DOCX 21 kb) [file 12916_2018_1107_MOESM3_ESM.docx]

## Additional file 3: CERQual Analysis

|  | Overall score:  1.0 (no major methodological flaws) 0.5 (some flaws) 0.1 (several significant flaws) | Comment | 1. Did the study design meet our inclusion criteria? | 2. Was the study context clearly described and appropriate? | 3. Was the method of data collection clearly described and valid? | | 4. Was the sampling method clearly described and appropriate? | 5. Was the method of analysis clearly described and appropriate? | 6. Was there evidence of researcher reflexivity? | 7. Were the conclusions supported by the data? |
| --- | --- | --- | --- | --- | --- | --- | --- | --- | --- | --- |
| Hindhede 2014 | 1.0 | Qualitative study examining the narratives of people engaged in diabetes prevention interventions. | Y | | Y | Y | Y | Y | N | Y |
| Greenhalgh 2015 | 1.0 | Medium sized qualitative study examining the narratives of south asian patients from a deprived area in london with a history of GDM | Y | | Y | Y | Y | Y | N | Y |
| Jallinoja 2008 | 0.5 | 30 individuals interviewed after intervention. Mixed social class, narrow age range 52-65. Ethnicity unknown. All 1yr after lifestyle intervention | Y | | Y | Y | N | Y | N | Y |
| Walker 2012 | 0.5 | Story eliciting narrative of 29 people a year after participating in lifestyle intervention to reduce diabetes risk. | Y | | Y | Y | N | Y | N | Y |
| Troughton 2008 | 0.1 | Small qualitative study on skewed sample, no clear theoretical approach, not contextualised in existing literature. | Y | | Y | Y | N | N | N | N |
| Satterfields 2003 (USA) | 0.1 | Story elliciting narrative of 235 people | Y | | Y | N | N | N | N | Y |
| Tang 2015 | 0.5 | Semi- structured interviews of 23 women with a previous history of gestational diabetes | y | | y | Y | N | Y | N | Y |
| Vlaar 2014 | 1.0 | 535 people recruited to RCT completed structued questionnaire with likert scales | Y | | Y | Y | Y | Y | N | Y |
| Kim 2007 | 0.5 | 217 affluent white women with a hx of GDM. High educational attainment and all had insurance. Computer assisted telephone survey or written survey | Y | | Y | Y | Y | Y | N | N |
| Kolb 2015 | 1.0 | 60 item MCQ survey performed in person or on telephone performed by 54 people enrolled in an RCT. | Y | | Y | Y | Y | Y | N | Y |
| Jones 2011 | 0.5 | 22 women GDM within the last 7 years. Well educated and employed. Mixed methods- quantitative measures, survey with scales, semi-structured interview. | Y | | Y | Y | N | Y | N | Y |
| Morrison Z 2014 | 0.5 | Semi-structured story telling interviews. 21 narrative interviews. | Y | | Y | Y | Y | N | N | Y |
| Penn 2015 | 0.5 | Semi-structured interviews with Pakistani women at high risk of diabetes | y | | y | y | N | Y | N | N |
| Morrison 2009 | 1.0 | Well-conducted survey of national database sample. Looked for predictors of returning for GTT post-partum. 36% response rate. | Y | | Y | Y | Y | Y | N | Y |
| Penn 2018 | 1.0 | 20 semi-structured interviews and one focus group | Y | | Y | Y | Y | Y | N | Y |

**CERQual Questions in Full**

1. Did the study design meet our inclusion criteria?
   • Cross-sectional quantitative
   • Cross-sectional mixed-methods
   • Qualitative (including qualitative evaluation component of RCT)
   • Longitudinal quantitative
   • Longitudinal mixed-methods
   • Case-control
2. Was the study context clearly described and appropriate?
   • Was the aim of the study and the research question(s) clearly stated or readily inferred?
   • Was the study design appropriate to address the research question?
3. Was the method of data collection clearly described and valid?
   • In questionnaire studies, was the instrument validated, and/or was its development clearly described and appropriate?
   • In observational studies, were the data source(s) robust?
   • In qualitative studies, were the methods clearly described and appropriate?
4. Was the sampling method clearly described and appropriate?
   • Were the participants and data sources clear?
   • Did the authors recognise the possibility of sampling bias and take steps to guard against it?
   • Was sample size and response rate adequate (for quantitative studies, to reach statistical significance; for qualitative studies, to reach saturation of key themes)?

1. Was the method of analysis clearly described and appropriate?
   • In quantitative studies, was there a clear and valid description of the analysis process, rigor of statistical tests employed and reported?
   • In qualitative studies, was there a clear and valid description of the analysis process and development and/or application of theory?
2. Was there evidence of researcher reflexivity?
   • Did the authors adequately consider how their own backgrounds and perspectives and/or the wider context of the study may have influenced the findings?
3. Were the conclusions supported by the data?
   • Did the data provide sufficient depth, detail, and richness?
   • Were the findings clearly stated and definitive?
   • Did the findings support the authors’ interpretations and conclusions?
   • Did the authors consider all potential alternative explanations of their findings?
