# Supplementary material for: How are health-related behaviours influenced by a diagnosis of pre-diabetes? A meta-narrative review
Source: BMC Med. 2018 Jul 27;16:121. doi: 10.1186/s12916-018-1107-6 (PMC6062879; doi:10.1186/s12916-018-1107-6)
Supplement: Supplementary file 4 — Detailed summaries of study findings. (DOCX 18 kb) [file 12916_2018_1107_MOESM4_ESM.docx]

## Additional file 4: Detailed Summaries of Study Findings

| **Author** | **Paper no** | **Author's evaluation of Pre-diabetes** | **Pre-diabetes and risk perception: participant views** | **Social-Cultural Themes Identified** |
| --- | --- | --- | --- | --- |
| Hindhede 2014 | 1,2 | Challenge categorisation, reports downplays role of social and material circumstances. The medical categorisation places problem outside political and ideological consideration. Results in the making of a 'new person'. | Accepted premise of condition and welcomed labelling. Diagnosis seen as an individual weakness which needed a response through regulation and control. | Social capital, cultural expectations, self-efficacy, material capital, cultural identity, self-control, social context |
| Greenhalgh 2015 | 3 | Acceptance of medical condition and its role as a risk factor for future diabetes development. Accept lifestyle change as a method for reducing risk. But emphasis on wider social cultural complexity. | Negative response to categorisation of GDM, continued in the post-partum period. Focus on future risk of diabetes absent as primary concern is wellbeing of child, ignoring their own health. | Cultural expectations, social context, economic insecurity, social capital, material circumstances, poor housing, health literacy, self-efficacy, cultural identity, self-control, environmental influences, health system |
| Jallinoja 2008 | 4 | Authors accept place the emphasis on individuals to be responsible for diabetes prevention. | Acceptance of the risk categorisation from participants and acceptance that individuals have responsibility for health. | Health literacy, self-control, responsibility, self-regulation |
| Walker 2012 | 5 | Accept premise of pre-disease with biomedical model and ability to prevent diabetes through lifestyle interventions. Two authors involved in design and quantitative evaluation of intervention. | Positively accepted risk categorisation of pre-diabetes and mathematical risk tool was well received. | Social capital, health literacy, self-control, responsibility. |
| Troughton 2008 | 6 | Accept premise of pre-disease placing it within the biomedical. Authors designed lifestyle interventions in RCTs. | Uncertainty with how categorisation sits with diabetes and what to do about it. Uncertain regarding the seriousness of pre-diabetes and uncertain as to how to take action. | social capital, self-efficacy, responsibility, cultural identity, regulation, health care system |
| Satterfield 2003 | 7 | Accept premise of pre-disease placing it within the biomedical model. | Perceived themselves to be at low risk, welcomed news that not diabetes yet. Thought categorisation was a useful tool but didn't think diabetes could be prevented. | social capital, acculturation, cultural responsibilities, economic insecurity, environmental influences, health system, self-efficacy, responsibility |
| Tang 2015 | 8 | Acceptance of medical condition and its role as a risk factor for future diabetes development. Accept lifestyle change as a method for reducing risk. Focus all on the individual no reflection on wider social structures. | 50% perceived themselves to be at high risk of diabetes development. Momentum to reduce risk of diabetes dissipated after pregnancy. Some uncertain and confused regarding the magnitude of association between pre-diabetes and diabetes. | social capital, traditional cultural roles, emotional barriers, material constraints, health literacy, health system, responsibility |
| Vlaar 2014 | 9 | Acceptance of medical condition and pre-condition. Report that south Asian people are high risk individuals and because these individuals have a strong family history of diabetes they should consider themselves 'more susceptible' than the general population. Lead author investigator in RCT. | 72.5% thought being south Asian caused diabetes, 88.9% though a family history caused diabetes, and 87% thought diabetes could be prevented. However, only 44.2% though they were at high risk of developing diabetes. | Not explored |
| Kim 2007 | 10 | Accept premise of condition which leads to 'pre-Diabetes and diabetes. | >90% knew that GDM increased risk of diabetes. Despite this only 41% thought they had a moderate chance and 16% high chance of developing diabetes | Health literacy, material circumstances, economic constraints, |
| Jones 2011 | 11 | Accept premise GDM and that this leads to pre-DM which linearly progresses to T2DM. | Knew GDM led to an increased risk of DM. Reported moderate- high chance of developing diabetes which increased to high chance if didn't change behaviour. Uncertainty with regards to inevitability and preventability of T2DM. | Control, self-efficacy, social context, material circumstances, cultural expectations, health literacy, cultural identity, self-surveillance |
| Morrison Z 2014 | 12 | Accept premise of the condition and authors architects of RCT. | Not explored, only two participants joined the trial to reduce risk of diabetes, others thought aim was to lose weight or provide information. | social context, cultural expectations, environmental influences, cultural responsibilities, self-efficacy, responsibility, health services, social capital |
| Penn 2015 | 13 | Accept premise of the condition and authors architects of intervention. | Didn't explore how people felt about the categorisation or risk perception. | social context, social capital, self-efficacy, emotional link to food, economic constraints, material circumstances, health literacy, cultural identity, control, self-surveillance |
| Kolb 2015 | 14 | Accept premise of the condition and authors architects of intervention. | All know what pre-diabetes was and that increased risk of diabetes. All perceived themselves at high risk of DM. | Social capital and support, health literacy, self-efficacy, self-regulation, responsibility, material circumstances |
| Morrison 2009 | 15 | Accept premise of condition and risk state. | Only 26% thought they were at high or very high risk of developing diabetes. 93% recall that GDM was a risk factor for T2DM. History of GDM not a strong influence for risk perception. No association between lifestyle factors and risk of diabetes. | Not explored |
| Penn 2018 | 16 | Accept premise of condition and the risk state | Didn’t explore how people felt about the diagnosis or the categorisation | Social support, peer support, cultural influences. |
